# The Defibrillation System of Basic Emergency Medical Technicians in Japan: A Comparison with Other Systems From a 14-Year Review of Out-of-Hospital Cardiac Arrest Reports

**DOI:** 10.2188/jea.11.29

**Published:** 2007-11-30

**Authors:** Miho Sekimoto, Mahbubur Rahman, Yosinori Noguchi, Kenji Hira, Takuro Shimbo, Tsuguya Fukui

**Keywords:** cardiac arrest, emergency medical services, defibrillation, out-of-hospital CPR, utstein style

## Abstract

Although seven years have passed after basic emergency medical technician (EMT) defibrillation-system was introduced in Japan, the overall survival for out-of-hospital cardiac arrest (OHCA) remains poor. We investigated factors leading to such an unanticipated result in Japan by comparing the data of OHCA in Japan with those in other countries. We obtained population-based OHCA data from three communities in Japan. We also performed a comprehensive literature and manual search to identify reports that included rates for incidence and survival or provided sufficient data for the calculation of these rates of OHCA. Statistical analysis was performed to compare survival and incidence rates between the communities. We identified 36 articles from 16 countries by a comprehensive literature search. There was no significant difference in incidence and survival rates among communities in Japan. Although the incidence rate of col lapse-witnessed OHCA with ventricular fibrillation (VF) was much lower in Japan than western countries, the proportions of survival from it were comparable to those. Basic-EMT defibrillation system in Japan has yielded excellent result in terms of the survival of VF cases. However, much lower proportion of VF to all cases is responsible for lower overall rates of survival from OHCA in Japan.

## INTRODUCTION

There is much controversy regarding the effectiveness of different emergency medical service (EMS) systems on out-of-hospital cardiac arrest (OHCA) because of wide variation in reported survival rates among medical centers, jurisdictions and countries^1^^-^^3^^)^. This variation is considered to be attributable to difference in the types of EMS system, proportion of bystander-initiated cardiopulmonary resuscitation (CPR), response time intervals of EMS providers, or geography of the communities in question.

The first program to resuscitate victims of OHCA began in Belfast in 1966^4^^)^. It has soon become popular around the world. Consequently new cadres of health professionals, paramedics, and emergency medical technicians have been trained to perform advanced cardiac life support in many countries. At the same time, advances in defibrillation (e.g., automatic external defibrillators) have expanded the scope of defibrillation from physicians, nurses, and paramedics to emergency medical technicians (EMTs). The defibrillation by basic EMTs proved to be effective by several randomized controlled trials and meta-analyses^5^^-^^8^^)^.

Before 1993, EMS personnel in Japan were only the basic life support providers who administered oxygen and CPR to victims of cardiac arrest. In 1993, the system was changed and emergency medical technicians specializing in defibrillation (EMT-Ds) were trained to perform airway control using laryngeal mask airway, vascular access, and defibrillation with semiautomatic external defibrillators.

The effectiveness of this early basic EMT defibrillation system in Japan, however, has been the topic of controversy. The Japanese Society for Emergency Medicine conducted a survey at 33 facilities supervised by their members, to compare the overall survival rates of OHCA before and after the introduction of early basic EMT defibrillation system. The survival rates (complete recovery) were 1.0% (28 out of 2887) in 1989 and 0.9% (12 out of 1278) in 1996, which revealed no improvement at all^9^^)^. However, it is difficult to draw a definitive conclusion from these data on the effectiveness of this new system, since there is no standardized or systematic comparative study in Japan.

The Utstein style, the approved guidelines for uniform reporting of data on OHCA^10^^)^, was developed in the early 1990s and enabled us to compare the outcome of OHCA and effectiveness of EMS systems among different communities and countries. The objective of this study is to compare outcomes of OHCA ever done in Japan with those in other countries according to the Utstein style.

## MATERIALS AND METHODS

### 1. Population-based data from three suburban communities in Japan

#### 1.1 EMS System in Japan

Japan has about 1,600 jurisdictions, all of which are covered by a uniform single-response EMS system. There are several exceptional communities where the physician staffed mobile intensive care unit make up the second tier. The number of EMS personnel is determined by law to be 1/1,000 population. Before 1993, EMS personnel in Japan were only the basic life support providers who administered oxygen and CPR to victims of cardiac arrest. In 1993, the system was changed and EMT-Ds were trained to perform airway control using laryngeal mask airway, vascular access, and defibrillation with semiautomatic external defibrillators. The total number of EMT-Ds has been increasing and reached 7,500 (about 6/100,000 population) by the end of 1998.

Dispatch of EMS is criteria-based, computer-aided, and performed by dispatchers who are full-time employees. Some dispatchers are trained to give instructions for bystander-initiated CPR over the telephone. In most cases of OHCA, EMT-Ds are the first to respond on site. Physicians’ decision is required by law to provide defibrillation to patients by EMT-Ds. Patients’ ECG is recorded and televised to physicians in charge to obtain permission of defibrillation.

The law obliges every jurisdiction of EMS system to report the annual incidence and outcome (return of spontaneous circulation and 1 month survival) of all cardiac and respiratory arrest victims to whom EMS personnel attempted resuscitation.

#### 1.2 Data Collection

Since this study did not involve any new intervention or procedure and the data collected here are part of routine quality assurance activities required for EMS systems in each community studied, we did not seek Institutional Review Boar’s approval for this study.

The OHCA data in the Utstein style were collected prospectively from three suburban communities: Akita (population, 316,000; area, 460 km^2^), Otsu (population, 306,000; area, 370 km^2^), and Izumo (population, 128,000; area, 440 km^2^). Data from Akita were collected in 1995-1998, those from Otsu in 1997-1998, and those from Izumo in 1998-1999. All of these communities have a uniform single-response EMS system. Cardiac arrest was defined to be the cessation of cardiac mechanical activity, confirmed by the absence of a detectable pulse, unresponsiveness, and apnea according to the Utstein style^10^^)^. All cardiac arrest cases confirmed by EMS personnel were included in this study, while cases with irreversible signs of death at the first contact were excluded. The causes of cardiac arrest were verified from the reported situation of collapse, medical history, clinical examinations, and autopsy reports. Outcome data, death or survival to discharge, were obtained from hospital records.

### 2. Literature Search

#### 2.1 Inclusion and Exclusion Criteria

We performed a comprehensive literature search and obtained population-based OHCA data in the Utstein style or a quasi-Utstein style. We identified articles published in peer-reviewed journals from January 1985 through December 1998 using a comprehensive MEDLINE database with the keywords of “heart arrest (MeSH),” “cardiac arrest,” “Utstein style,” “out-of-hospital,” “prehospital,” “emergency medical service (MeSH),” and “prognosis (MeSH)”. All published primary studies, but no unpublished studies or abstracts, were included in the analysis. We then conducted a manual search of the references cited in published reports or previous reviews^1^^-^^3^^)^. Only articles published in English were analyzed. Scientific articles regarding OHCA in Japan in the Utstein style were obtained by the Japanese computer-based on-line database “IGAKU CHUOH ZASSHI” using the same keywords stated above.

#### 2.2 Data Extraction

The following variables, when available, were extracted from each study.

(1) Year of investigation
(2) Population and area served by each EMS system
(3) Types of EMS personnel (conforming to the definitions of Nichol et al.^10^^)^)
(4) Annual number of OHCA patients on whom EMS personnel attempted CPR (CPR-attempted OHCA)
(5) Annual number of CPR-attempted OHCA cases with presumed cardiac etiology (cardiac-OHCA)
(6) Annual number of cardiac-OHCA survivors
(7) Annual number of cardiac-OHCA cases in which the initial electrocardiographic rhythm was ventricular fibrillation (VF-OHCA)
(8) Annual number of VF-OHCA survivors
(9) Annual number of CPR-attempted, bystander-witnessed OHCA cases in which the initial electrocardiographic rhythm was VF (witnessed-VF OHCA)
(10) Annual number of witnessed-VF OHCA survivors

From the abstracted data, we then calculated the OHCA incidence rates (annual number of OHCA cases per population served), the incidence rates for OHCA survivors (annual number of OHCA survivors per population served), and survival rates (number of OHCA survivors per number of OHCA cases) for each type of EMS system and compared them among communities and countries.

### 3. Statistical Analysis

The annual incidence rate of OHCA for each category was not standardized by age and sex. The 95% confidence intervals of incidence rates were calculated by using the exact method of the Poisson distribution, and those for survival rates were calculated by using the exact method of the binomial distribution.

We calculated summary estimates of survival rates for cardiac-OHCA and witnessed-VF in Japan; homogeneity across data was tested using the Woolf chi-square statistics. To calculate the summary estimates, each data was weighted by its sample size. The customary value of a two-sided p-test (p<0.05) was used. All statistical analyses were performed using the Stata Ver. 6.0 statistical package (Stata Corp. College station, TX).

## RESULTS

### 1. Overview of OHCA in Japan

Literature search identified only one population-based study in Utstein style from Japan that was conducted in Funabashi (population, 540,000; area, 86 km^2^)^11^^)^ in 1993-1997. Funabashi EMS is two-tiered system; i.e. for each call judged as OHCA, a physician staffed mobile intensive care unit and the standard ambulance are dispatched simultaneously. We compared the data from four communities (one by literature search and three by primary data), the incidence rates of CPR-attempted OHCA ranged from 34 to 49/100,000/year. About the half of the CPR-attempted OHCA cases were clearly of noncardiac origin, such as trauma, poisoning, suffocation, suicide, respiratory failure, stroke, and terminal illness. The CPR-attempted OHCA with explicit cardiac etiology accounted for only a half of the remainders. The incidence rates of cardiac-OHCA according to the Utstein definition ranged from 18 to 26/100,000/year, and those of witnessed-VF OHCA 2.6 to 3.6 (10% to 18% of cardiac-OHCA). The incidence rates of the survivors of cardiac-OHCA ranged from 0.6 to 2.0/100,000/year, 50% to 100% of these were accounted for by witnessed-VF OHCA cases.

The proportion of survival from cardiac-OHCA ranged from 5.4% to 10% and those of witnessed-VF OHCA 21% to 40%. There was no statistically significant difference in these proportions among the four communities. The incidence rates of CPR-attempted OHCA and cardiac-OHCA in Funabashi were slightly lower than those in the other three communities; however, the incidence rate of witnessed-VF was almost equal to those in the other three communities. The summary estimate for the proportion of survival from cardiac-OHCA in Japan was 6.7% ( $\chi_{\text{h}}^{2}<4.9$ , p<0.18, 95% CI 5.0% to 8.4%) and that from the witnessed-VF OHCA in Japan was 28.1% ( $\chi_{\text{h}}^{2}<4.5$ , p<0.22,95% CI 0.4% to 35.9%).

### 2. International Comparison of the Outcomes of OHCA

We identified 35 articles: 12 articles from the United States^12^^-^^23^^)^, 3 each from Finland^24^^-^^26^^)^ and Germany^27^^-^^29^^)^, 2 each from Canada^30^^, ^^31^^)^, the Netherlands^32^^, ^^33^^)^, New Zealand^34^^, ^^35^^)^, and the United Kingdom^36^^, ^^37^^)^, and one apiece from Sweden^38^^)^, Slovenia^39^^)^, Italy^40^^)^, France^41^^)^, Australia^42^^)^, Israel^43^^)^, Hong Kong^44^^)^, and Taiwan^45^^)^. The data extracted from these articles are shown in Table 1 along with the four sets of data from Japan.

**Table 1.** Global comparison of incidence and survival rate for OHCA. Based on MEDLINE and IGAKU-CHUOH-ZASSHI databases* published during 1985-98.

Country	Location	Duration	Population (in 10,000)	Types of EMS personnel	IR of CPR- Attempted OHCA	Cardiac-OHCA			VF-OHCA			Witnessed-VF OHCA		
		
						IR	IR of Survivors	Survival Rate (%)	IR	IR of Survivors	Survival Rate (%)	IR	IR of Survivors	Survival Rate (%)
U.S.A.	King County, Washington	1976-83	70	BLS+BLS-D+ALS	—	39 (37 - 41)	7.1 (6.4 - 7.9)	18 (16 - 20)	—	—	—	—	—	—
	San Juan Island, Pacific Northwest	1977-94	0.5	BLS-D+ALS	114 (93 - 138)	91 (72 - 113)	19 (11 - 31)	21 (13 - 32)	—	—	—	46 (33 - 62)	19 (11 - 31)	43 (27 - 59)
	Minnesota	1982-84	20	BLS+BLS-D+ALS	145 (134 - 158)	128 (117 - 140)	4.0 (2.3 - 6.5)	3.1 (1.8 - 5.0)	—	—	—	—	—	10.3 (5.2 - 18)
	Monroe County, New York	1986-87	71	BLS+ALS	65 (59 - 71)	48 (43 - 53)	34 (2.2 - 5.0)	7.0 (4.6 - 10)	—	—	—	—	—	—
	New York City	1986-93	13	BLS+BLS-D+ALS	47 (43 - 51)	40 (36 - 44)	0.7 (0.3 - 1.4)	17 (0.7 - 3.5)	—	—	—	—	—	—
	Pennsylvania	1987-91	15	BLS+ALS	88 (81 - 95)	71 (65 - 77)	3.7 (2.5 - 5.4)	5.3 (3.5 - 7.5)	34 (30 - 39)	2.9 (1.8 - 4.4)	8.5 (5.4 - 13)	—	—	—
	Chicago	1987	270	ALS	146 (142 - 151)	119 (115 - 124)	2.0 (1.5 - 2.7)	1.7 (1.3 - 2.2)	28 (26 - 30)	1.1 (0.7 - 1.5)	3.8 (2.6 - 5.5)	—	—	—
	Houston, Texas	1988-89	180	—	68 (65 - 72)	55 (51 - 58)	5.4 (4.4 - 6.6)	9.8 (8.1 - 12)	23 (21 - 25)	4.3 (3.4 - 5.4)	19 (15 - 23)	9.6 (8.2 - 11)	1.4 (0.9 - 2.1)	15 (10 - 21)
	Memphis, Tennessee	1989-92	61	BLS+BLS-D+ALS	—	53 (50 - 56)	4.2 (3.4 - 5.2)	8.0 (6.4 - 9.7)	26 (24 - 28)	3.1 (2.4 - 4.0)	12 (9.5 - 15)	—	—	—
	Oakland, Michigan	1989-93	100	BLS+ALS	—	63 (61 - 65)	—	—	—	—	—	13 (12 - 14)	—	—
	York Adams, Pennsylvania	1988-89	41	BLS+ALS	87 (81 - 94)	73 (67 - 79)	4.4 (3.1 - 6.1)	6.0 (4.2 - 8.2)	33 (29 - 37)	3.3 (2.2 - 4.8)	10 (6.7 - 14)	—	—	—
	New York City	1990-91	733	BLS-D+ALS	89 (85 - 92)	64 (61 - 66)	1.4 (1.1 - 1.9)	2.2 (1.7 - 2.9)	—	—	—	13 (12 - 14)	1.0 (0.7 - 1.4)	7.9 (5.7 - 11)
Canada	Ontario	1986-87	150	BLS	—	50 (47 - 54)	1.1 (0.6 - 1.7)	2.1 (1.2 - 3.4)	—	—	—	—	—	—
	Ontario	1988-89	150	BLS-D	—	50 (47 - 54)	1.5 (0.9 - 2.2)	2.9 (1.8 - 4.4)	—	—	—	—	—	—
	Hamilton-Wentworth, Ontario	1990-91	45	BLS+BLS-D+ALS	—	67 (59 - 75)	2.7 (1.4 - 4.7)	4.0 (2.1 - 7.0)	26 (22 - 31)	2.5 (1.2 - 4.4)	9.5 (4.8 - 16)	—	—	—
Finland	Helsinki	1987	50	BLS+Ph	53 (47 - 60)	48 (42 - 55)	—	—	—	—	—	29 (24 - 34)	8.2 (5.9 - 11)	29 (21 - 37)
	Helsinki	1991-92	51	BLS-D+ALS+Ph	55 (49 - 62)	47 (41 - 53)	—	—	—	—	—	24 (20 - 28)	6.5 (4.5 - 9.1)	28 (20 - 36)
	Helsinki	1992-93	51	BLS-D+ALS+Ph	63 (57 - 71)	61 (54 - 68)	—	—	—	—	—	26 (20 - 29)	8.0 (5.8 - 11)	32 (24 - 40)
	Helsinki	1994	52	BLS-D+ALS+Ph	67 (60 - 74)	46 (40 - 52)	9.7 (7.2 - 12.8)	21 (16 - 27)	—	—	—	24 (20 - 29)	7.6 (5.4 - 10)	31 (23 - 40)
Germany	Bonn	1989-92	24	BLS+BLS-D+ALS	54 (50 - 59)	48 (44 - 53)	7.7 (6.1 - 9.7)	16 (13 - 20)	22 (19 - 25)	5.4 (4.0 - 7.1)	25 (19 - 31)	15 (13 - 18)	5.0 (3.7 - 6.6)	34 (26 - 42)
	Mainz	1991-92	18	BLS+BLS-D+ALS	90 (79 - 92)	84 (73 - 96)	7.5 (4.5 - 12)	9.0 (5.5 - 14)	35 (28 - 43)	5.9 (3.3 - 9.7)	17 (9.6 - 26)	21 (16 - 28)	5.2 (2.8 - 8.8)	24 (14 - 38)
	Gottingen	1994-95	16	ALS+Ph	84 (73 - 98)	71 (60 - 84)	16 (11 - 22)	22 (16 - 30)	—	—	—	23 (17 - 30)	9.8 (6.1 - 15)	44 (30 - 59)
the Netherlands	Leiden	1989-92	20	BLS+ALS	—	45 (40 - 50)	6.1 (4.4 - 8.3)	14 (10 - 18)	29 (25 - 34)	5.8 (4.2 - 7.9)	20 (15 - 26)	—	—	—
	Amsterdam	1995-97	130	ALS	46 (43 - 48)	37 (35 - 40)	4.8 (4.0 - 5.6)	13 (11 - 15)	23 (21 - 25)	—	—	18 (17 - 20)	—	—
Sweden	Goteborg	1980-92	43	BLS+BLS-D+ALS	64 (62 - 67)	54 (52 - 56)		—	—	—	—	18 (17 - 19)	—	—
U.K.	South Glamorgan	1989-92	41		84 (78 - 89)	61 (57 - 66)	3.9 (2.8 - 5.2)	6.3 (4.6 - 8.4)	20 (18 - 23)	—	—	17 (15 - 20)	3.6 (2.6 - 4.9)	21 (16 - 28)
	Edinburgh	1994-95	75	BLS-D+ALS	55 (49 - 60)	37 (33 - 41)	2.1 (1.2 - 3.5)	5.8 (3.4 - 9.3)	—	—	—	—	—	—
Slovenia	Ljubljana	1995-97	40	BLS+ALS	38 (35 - 42)	28 (25 - 32)	2.0 (1.3 - 3.0)	5.6 (3.4 - 8.7)	10 (8.4 - 12)	1.6 (1.0 - 2.5)	12 (7.1 - 20)	9.6 (7.9 - 12)	1.3 (07 - 2.1)	13 (7.6 - 21)
Italy	Friuli Venezia Giulia region	1994	94	BLS+ALS+Ph	47 (42 - 51)	37 (33 - 41)	2.5 (1.6 - 3.7)	6.7 (4.3 - 9.9)	—	—	—	—	—	—
France	Saint-Etienne	1991-92	57	BLS-D+Ph	41 (36 - 47)	20 (16 - 24)	1.4 (0.6 - 2.8)	7.1 (3.1 - 14)	7.9 (5.8 - 11)	1.2 (0.5 - 2.5)	16 (6.5 - 30)	6.8 (4.9 - 9.3)	1.2 (0.5 - 2.5)	18 (7.5 - 34)
N.Z.	Auckland	1983	94	BLS+ALS	43 (39 - 48)	26 (23 - 29)	4.3 (3.1 - 5.8)	17 (12 - 22)	25 (23 - 27)	4.8 (4.0 - 5.7)	19 (17 - 23)	—	—	—
	Auckland	1991-93	94	BLS-D+ALS	42 (40 - 44)	38 (36 - 41)	4.8 (4.0 - 5.7)	13 (11 - 15)	—	4.0 (2.8 - 5.5)	—	19 (18 - 21)	3.2 (2.5 - 3.9)	16 (13 - 20)
Australia	Ipswich & West Moreton Shires	1985-89	9	BLS+BLS-D+ALS	—	51 (44 - 58)	4.0 (2.3 - 6.4)	7.9 (4.7 - 12.4)	24 (20 - 29)	3.5 (2.0 - 5.8)	15 (8.4 - 23)	—	—	—
Israel	Magen David Adom	1984-85	329	BLS+ALS+Ph	—	46 (44 - 47)	3.0 (2.6 - 3.5)	6.6 (5.7 - 7.6)	14 (13 - 15)	2.2 (1.8 - 2.6)	16 (13 - 18)	—	—	—
China	Hong-Kong	1990-92	45	BLS	23 (21 - 26)	12 (10 - 14)	0.4 (0.1 - 0.9)	3.0 (0.8 - 7.4)	—	—	—	—	—	—
Taiwan	Taipei	1992-93	270	BLS	26 (24 - 28)	20 (18 - 22)	0.3 (0.1 - 0.6)	1.4 (0.5 - 3.1)	—	—	—	—	—	—
Japan	Funabashi	1993-97	54	BLS+BLS-D+Ph	34 (31 - 36)	18 (16 - 20)	0.6 (0.3 - 1.0)	5.4 (3.8 - 8.2)	—	—	—	2.7 (2.0 - 3.5)	0.6 (0.3 - 1.0)	21 (11 - 33)
	Akita*	1995-98	31	BLS+BLS-D	49 (45 - 53)	20 (17 - 22)	2.0 (1.3 - 2.9)	10.0 (6.6 - 15)	4.4 (3.3 - 5.7)	1.6 (1.0 - 2.4)	36 (24 - 50)	3.6 (2.6 - 4.8)	1.4 (0.8 - 2.3)	40 (26 - 56)
	Otsu*	1997-98	30	BLS+BLS-D	47 (42 - 53)	26 (22 - 30)	1.6 (0.8 - 3.0)	6.3 (3.1 - 11)	3.6 (2.3 - 5.4)	1.1 (0.5 - 2.4)	32 (14 - 55)	2.6 (1.5 - 4.2)	0.8 (0.3 - 1.9)	31 (11 - 59)
	Izumo*	1998-99	13	BLS+BLS-D	40 (31 - 49)	20 (15 - 27)	2.0 (0.5 - 5.1)	9.1 (1.1 - 29)	5.9 (3.1 - 10)	1.5 (0.3 - 4.3)	25 (5.5 - 57)	3.5 (1.4 - 7.1)	1.0 (0.1 - 3.6)	29 (3.7 - 71)

* Manual data from three settings in Japan were also included

Types of EMS : BLS=Basic Life Support Providers, BLS-D=Basic Life Support Providers with defibrillation, ALS=Advanced Life Support Providers, Ph=Physician

IR ; Annual incidence rate for 100,000 populations

CPR-attempted OHCA : OHCA in which CPR were attempted

Cardiac-OHCA : CPR-attempted OHCA of presumed cardiac etiology

VF-OHCA : Cardiac-OHCA with ventricular fibrillation as first cardiac rhythm

Witnessed-VF OHCA : collapse-witnessed cardiac-OHCA with VF as first cardiac rhythm

#### 2.1 CPR-attempted OHCA

The data from journal articles showed wide variation in the incidence rates of CPR-attempted OHCA among countries and communities. One extreme was as high as 145/100,000/year as reported from Minnesota^14^^)^ and Chicago^18^^)^, and the other as low as 23/100,000/year as reported from Hong Kong^44^^)^. The incidence rates of CPR-attempted OHCA in Asian countries including Japan ranged from 23/100,000/year to 49/100,000/year, much lower than those in western countries.

#### 2.2 Cardiac-OHCA

**Figure 1.**
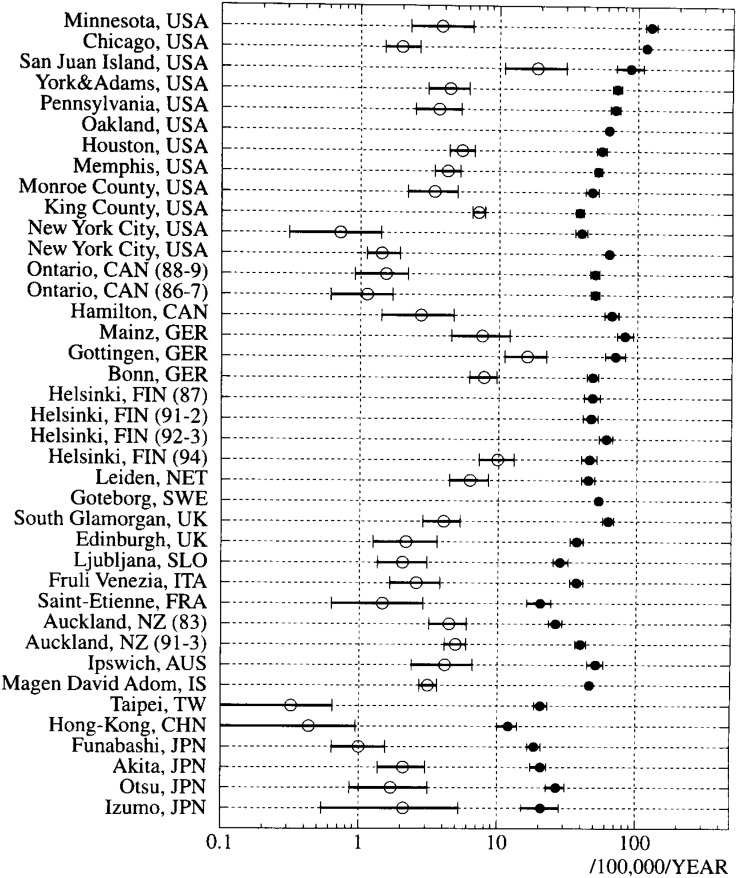
Incidence rates of cardiac-OHCA and its survivors (by logarithmic scale, annual rate per 100,000 population). The closed circles represent the incidence rates of cardiac-OHCA, and the open circles represent those of the survivors from cardiac-OHCA. Bars: 95% confidence intervals. USA: the United States, CAN: Canada, GER: Germany, FIN: Finland, NET: the Netherlands, SWE: Sweden, UK: the United Kingdom, SLO: Slovenia, ITA: Italy, FRA: France, NZ: New Zealand, AUS: Australia, IS: Israel, TW: Taiwan, CHN: China, JPN: Japan.

The annual incidence rates of cardiac-OHCA and its survivors are shown in Figure 1, revealing great regional differences. The incidence rates tend to be higher in Finland, Sweden, Germany, and North America, where morbidity from coronary heart disease (CHD) is high^46^^)^, and lower in France, Italy, and East Asia including Japan.

The community with the highest incidence rate of survivors for cardiac-OHCA was San Juan Islands, North Pacific, U.S. (19/100,000/year)^13^^)^ and the community with the lowest rate was Taiwan (0.3/100,000/year)^45^^)^. San Juan Islands also had a high incidence of cardiac-OHCA while Taiwan had a low incidence of cardiac-OHCA. The incidence rates of the survivors in Japan were not much different from those in western countries except for Finland, Germany, and San Juan Islands.

**Figure 2.**
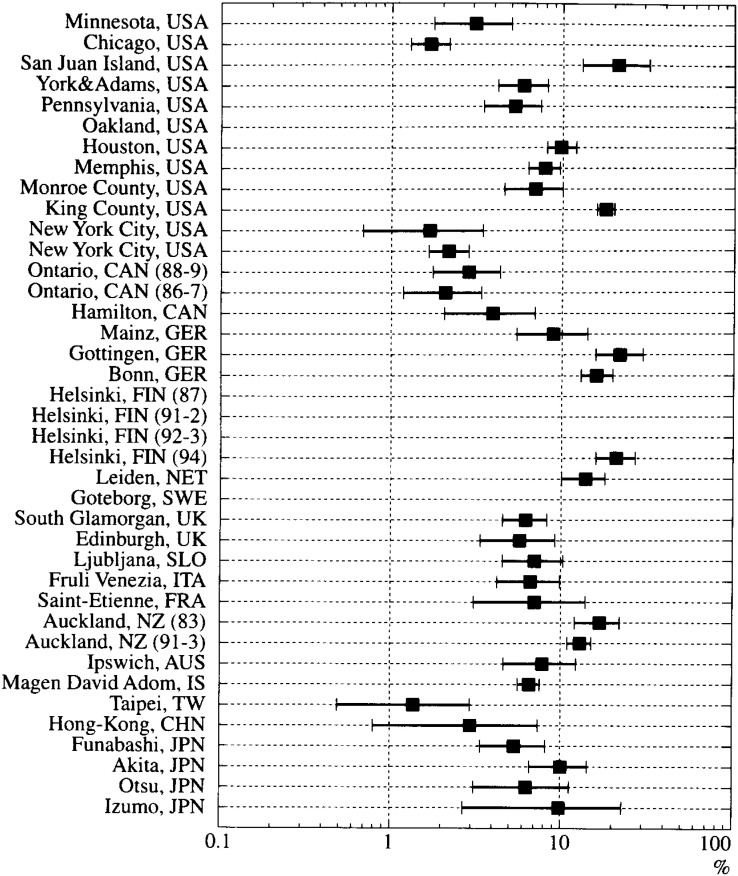
Proportion of survival from cardiac-OHCA. Bars: 95% confidence intervals. USA: the United States, CAN: Canada, GER: Germany, FIN: Finland, NET: the Netherlands, SWE: Sweden, UK: the United Kingdom, SLO: Slovenia, ITA: Italy, FRA: France, NZ: New Zealand, AUS: Australia, IS: Israel, TW: Taiwan, CHN: China, JPN: Japan.

The proportions of survival from cardiac-OHCA are shown in Figure 2. The proportion ranged from 1.4% to 22%. Although the range of those reported from a variety of communities in the United States was wide, from 1.7% (New York^16^^)^ and Chicago^18^^)^) to 21% (San Juan Islands^13^^)^), the majority were around 10%. On the other hand, reports from Asian countries showed dismal figures: 3.0% in Hong Kong^44^^)^ and 1.4% in Taiwan^45^^)^. However, the figures for communities in Japan ranged from 5.4% to 10.0%, similar to those of the majority of western countries.

#### 2.3 VF-OHCA

We identified sixteen investigations which reported the incidence rates of VF-OHCA^17^^-^^20^^, ^^22^^, ^^27^^, ^^28^^, ^^31^^, ^^32^^, ^^34^^, ^^35^^, ^^39^^, ^^41^^-^^43^^)^, ranging from 7.9/100,000/year for Saint-Etienne, France^41^^)^ to 34.4/100,000/year for Pennsylvania, U.S.^17^^)^. Five investigations from the United States revealed smaller intercommunity variation in the incidence of VF-OHCA than that in cardiac-OHCA. For example, the incidence rate of cardiac-OHCA in Chicago, Illinois, was more than double that in Memphis, Tennessee^18^^, ^^20^^)^. However, the incidence rate of VF-OHCA in Chicago was 27.9/100,000/year (95% CI, 26.0 to 30.0) and that of Memphis was 25.7/100,000/year (95% CI, 23.6 to 28.0), with no significant difference between these two communities. Unfortunately, there was no report from Asian communities other than Japan in this regard. The incidence rates of VF-OHCA in Japan, ranging from 3.6 to 5.9/100,000/year, were below the lowest rates for western countries.

The incidence rates of the survivors from VF-OHCA as reported in the articles ranged from 1.1/100,000/year to 5.9/100,000/year with the proportion of survival from VF-OHCA from 3.8% to 24.8%. The proportions of survival from VF-OHCA in Japan (25% to 36%) were comparable to those in western countries.

#### 2.4 Witnessed VF-OHCA

**Figure 3.**
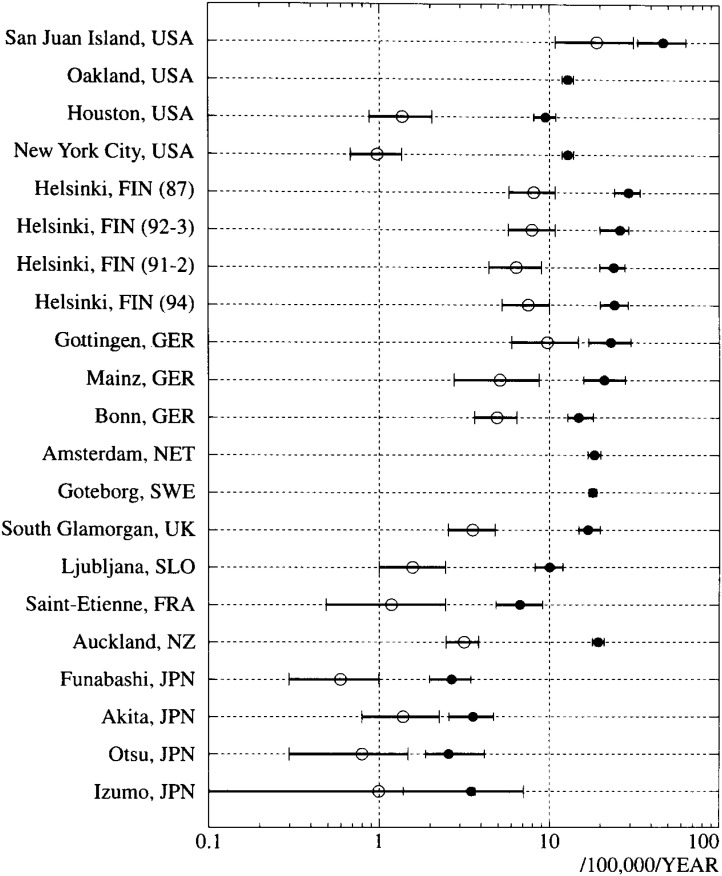
Incidence rates of witnessed VF-OHCA and those of its survivors (by logarithmic scale, annual rate per 100,000 population). The closed circles represent the incidence rates of witnessed VF-OHCA, and the open circles represent those of the survivors from witnessed VF-OHCA. Bars: 95% confidence intervals. USA: the United States, GER: Germany, FIN: Finland, NET: the Netherlands, SWE: Sweden, UK: the United Kingdom, SLO: Slovenia, FRA: France, NZ: New Zealand, JPN: Japan.

**Figure 4.**
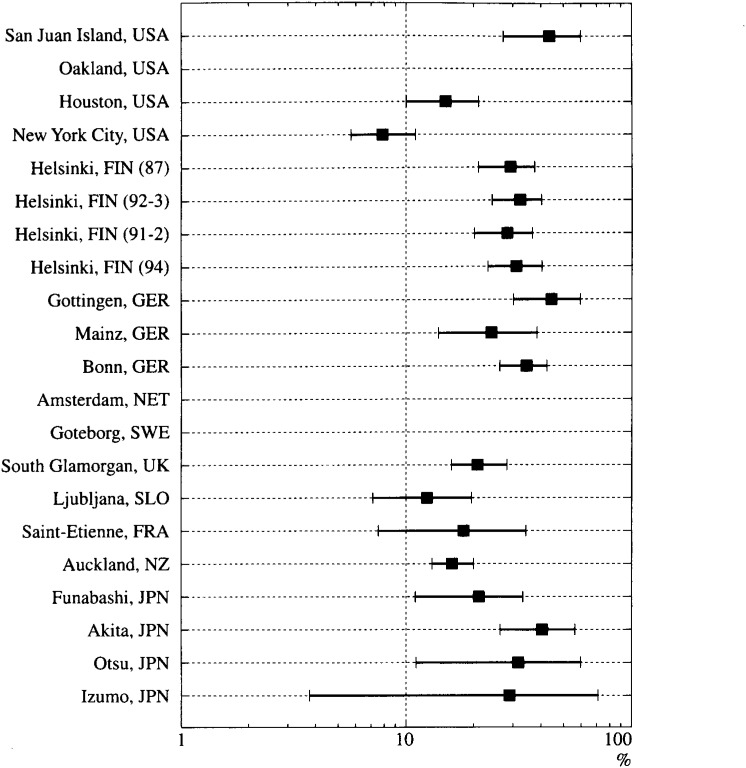
Proportion of survival from witnessed VF-OHCA. Bars: 95% confidence intervals. USA: the United States, GER: Germany, FIN: Finland, NET: the Netherlands, SWE: Sweden, UK: the United Kingdom, SLO: Slovenia, FRA: France, NZ: New Zealand, JPN: Japan.

The annual incidence rates of witnessed VF-OHCA and those for the survivors are shown in Figure 3. The incidence rates in western countries ranged from 6.8/100,000/year in Saint-Etienne to 46/100,000/year in San Juan Islands, while in Japan they ranged from 2.6/100,000/year to 3.6/100,000/year. The witnessed-VF OHCA cases accounted for 17% to 50% of cardiac-OHCA in the United States, 42% to 60% in Helsinki, Finland, 25% to 32% in Germany, 33% in Goteborg, Sweden, 28% in Glamorgan, U.K., 34% in Ljubljana, Slovenia, 34% in Saint-Etienne, France, and 50% in Auckland, N.Z. The proportion of survival from witnessed VF-OHCA in Japan (Figure 4), ranging from 21% to 51%, were comparable to the results of western countries.

## DISCUSSION

Survival among cases of OHCA is still rare in most countries. However, recent studies of EMS systems in Seattle and King County have shown remarkably high proportion of survival compared with other countries or communities^6^^, ^^12^^, ^^47^^-^^54^^)^, i.e., the proportions of survival being 16% from CPR-attempted OHCA, 18% from cardiac-OHCA, 30% from VF-OHCA, and 34% from witnessed VF-OHCA^55^^)^.

Many hospital-based studies on OHCA in Japan, in contrast to only a few population-based studies, demonstrated that the proportion of survival from CPR-attempted OHCA ranged from 2.8% to 4.4%^56^^-^^64^^)^ before the start of the new emergency system in 1993 and 1.9% to 6.1% afterwards^57^^, ^^58^^, ^^60^^-^^66^^)^. However, it is difficult to assess the impact of the new system by comparing the results of these studies, since such comparison is susceptible to a variety of biases, e.g. a lack of standard terminology, methodological differences, definition inconsistencies, and a variety of formats used in reporting the outcomes. The Utstein style was proposed as a solution to these problems^10^^)^. This style focuses on OHCA and includes a glossary of terms, a template for reporting data from resuscitation studies to ensure comparability, definitions for time points and time intervals related to CPR, definitions of individual clinical items and outcomes that should be included in reports, and recommendations for the description of EMS systems. Although most western countries have already adopted this style, Japan has seen its introduction in only a few communities.

The introduction of basic EMT defibrillation system in Japan may have been ineffective for the following reasons: (1) a lower incidence of OHCA caused by coronary heart disease (CHD) and a higher incidence of OHCA from a noncardiac origin compared with those in western countries^67^^, ^^68^^)^; (2) a low incidence of VF^60^^, ^^67^^-^^70^^)^; (3) a low incidence of bystander-initiated CPR before the arrival of EMS personnel^71^^-^^76^^)^; and (4) longer intervals from collapse to defibrillation because of the legal requirement of physicians’ determination to provide defibrillation.

Although there was some variation in EMS systems among four communities in Japan (single-response system for Akita, Otsu and Izumo, and two-tiered system for Funabashi), no marked difference was observed in the incidence rate and the ratio of survival for OHCA. Our extensive literature review have revealed very low incidence rates of cardiac-OHCA, especially that of witnessed-VF, in East Asia including Japan. Witnessed-VF cases accounted for less than 10% of all CPR-attempted OHCA cases in Japan. Moreover, the incidence rate of witnessed-VF in Japan was equivalent to only 20% of the highest incidence rates in western countries and accounted for only 5-10% of all CPR-attempted OHCA. Ironically, the annual number of VF-OHCA per 100,000 is lower than the number of EMT-Ds per 100,000 in Japan.

One of the reasons for this phenomenon is the low CHD morbidity in Japan. The World Health Organization’s MONICA Project, which tracked death cases due to CHD, has made an international comparison on the incidences of coronary events^46^^)^. It showed that age-standardized annual event rates in men aged 35 to 64 covered a twelve-fold range from 915/100,000/year for North Karelia, Finland, down to 76/100,000/year for Beijing, China. Although Japan did not participate in the MONICA Project, similar diagnostic criteria were used in monitoring coronary event rates. In this context, the data showed that the coronary event rate was 57/100,000/year for middle-aged men in Japan^77^^)^, almost comparable to that reported from Beijing, China. Other studies in Japan also showed low incidence of CHD compared with western countries^78^^, ^^79^^)^.

Previous studies demonstrated that collapse-to-EMS response intervals and presence of bystander-initiated CPR could affect the incidence of VF at EMS arrival on the scene^80^^-^^83^^)^. The low incidence of VF in Japan may be attributable to low incidence of bystander-initiated CPR and late EMS response. The proportions of bystander-initiated CPR among cardiac-OHCA ranged from 30% to 50% in Akita, Otsu, and Izumo, probably due to recent active campaigns that encouraged bystander-initiated CPR as well as telephone-assisted CPR, which were comparable to those in western countries. Moreover, although the proportion of bystander-initiated CPR was only 5.4% in Funabashi, it yielded no statistical difference in incidence rate of witnessed-VF OHCA compared with other three communities. The mean EMS response times in the studied communities (7.0 ± 3.0 min in Akita, 7.1 ± 4.7 min in Otsu, and 5.3 ± 3.5 min in Izumo) were not different from those reported from other countries^84^^)^.

The proportions of survival from witnessed-VF in Japan (average 28.1%, ranging from 21% to 51%) were not lower than those in western countries. Eisenberg suggested that the best possible proportion of survival from witnessed-VF should be about 30%^6^^)^. According to that ideal figure, the early basic EMT defibrillation system in Japan can be said to have already attained excellent results. However, the low proportion of witnessed-VF cases to all CPR-attempted OHCA cases is responsible for the considerably low overall survival rate in Japan.

The three major factors affecting the incidence of cardiac-OHCA are (1) the incidence rate of cardiac disease; (2) the diagnostic criteria of heart disease; and (3) the case selection for CPR by EMS personnel. The incidence rate of cardiac disease varies with age, race, socioeconomic status, and the coronary risk factors in each population. Unfortunately, of these factors, only age is reported consistently in the literature on cardiac arrest.

The diagnostic criteria for heart disease vary among centers and communities, making it one of the most inconsistent factors in the Utstein template. The Utstein style classifies OHCA with unknown origin as cardiac-OHCA. However, because autopsy is seldom performed on the victims of OHCA in Japan, this definition is highly likely to cause misclassification of cardiac-OHCA. In fact, the current data showed that only one of every three CPR-attempted OHCA cases was explicitly diagnosed as having cardiac etiology. Some investigations in Japan reported that only 38% to 56% of sudden death cases were ascertained to be of cardiac origin as confirmed by autopsy^85^^-^^87^^)^. Since the prognosis of OHCA with noncardiac origin is different from that with cardiac origin, misclassification would distort the apparent effectiveness of EMS systems.

The case selection for CPR by EMS personnel could also have great influence on the survival rates. The investigations in the United States showed that the incidence rates of cardiac-OHCA covered a three-fold range from 39/100,000/year to 128/100,000/year; however, there was little difference in the incidence rates of VF-OHCA among these communities, which suggests inconsistency in the case selection to perform CPR by EMS personnel and selection process of cardiac-OHCA. With more liberal criteria for selecting CPR attempted OHCA and cardiac-OHCA cases, survival rate would become lower. In Japan, the incidence rates of cardiac-OHCA were not significantly different among the communities, which suggested homogeneity of disease structure and case selection.

Witnessed-VF of presumed cardiac etiology is the optimum condition for survival and the proportion of it to all OHCA cases is likely to determine the overall survival rates. In general, the areas with a high incidence of witnessed-VF also had high coronary event rates, and therefore the favorable results of the introduction of basic EMT defibrillation system could be easily anticipated in these areas. On the other hand, in areas with a low incidence of witnessed-VF (South Europe and East Asia including Japan), the incidence rate of OHCA with cardiac origin is very low, which leaves little opportunity for the early basic EMT defibrillation system to lead better results.

Thus our current study demonstrated relatively excellent results of basic-EMT defibrillation system in Japan. However, these results do not necessarily represent the actual state of EMS system in all over Japan, since the communities studied are rather exceptional regions in the country with high incidence of bystander-initiated CPR and high conscience of both citizens and EMS personnel. Although the Utstein style has already been widely adopted in western countries^88^^)^, it has been utilized in only a few communities in Japan where voluntary EMS personnel collect it as part of their routine quality assurance activities.

There is a belief among epidemiologists, i.e., once a new programme has been started as a service to a population without prior evaluation, it is no longer possible to evaluate the programme in that particular population. If it was true, we can no longer evaluate the effectiveness of basic-EMT defibrillation system in Japan, since we have no standardized OHCA data prior to introduction of it. However, there still remains the way to locate the quality of EMS system in Japan by adopting the Utstein style to summarize the OHCA data and compare it with those in other countries as well as within the country. In this regard, every jurisdiction of EMS system in Japan should collect its own population-based OHCA data in a standardized format. At present, the Utstein style is the only such a method of data collection which enable us to make intrasystem and intersystem comparison of the performance of EMS in terms of the survival from cardiac arrest. Moreover, the adoption of this style would raise the morale of each EMS personnel and contribute to the quality assurance of EMS system in Japan.
